# Effects of Contextual Variables on Match Load in a Professional Soccer Team Attending to the Different Season Periods

**DOI:** 10.3390/s24020679

**Published:** 2024-01-21

**Authors:** Rodrigo dos Santos Guimarães, Tomás García-Calvo, Javier Raya-González, José C. Ponce-Bordón, Pedro Fatela, David Lobo-Triviño

**Affiliations:** 1 Faculty of Sport Sciences, University of Extremadura, Avenida de la Universidad S/N, 10003 Caceres, Spain; rodossant@alumnos.unex.es (R.d.S.G.); jrayag@unex.es (J.R.-G.); joponceb@unex.es (J.C.P.-B.); davidlt@unex.es (D.L.-T.); 2 Faculty of Human Kinetics, University of Lisbon, Estr. da Costa, 1495-751 Cruz Quebrada, Portugal; pedrofatela@fmh.ulisboa.pt

**Keywords:** workload, football, team sports, monitoring, GPS

## Abstract

This study aimed to analyze the effects of contextual variables (i.e., match location and match outcome) and season periods on match load (i.e., internal and external load) in professional Brazilian soccer players. Thirty-six professional players from the same soccer team participated in this study. The season was split into four phases: matches 1–16 (i.e., Phase 1 = P1); matches 17–32 (i.e., Phase 2 = P2); matches 33–48, (i.e., Phase 3 = P3); matches 49–65 (i.e., Phase 4 = P4). Considering match outcome, when the team wins, Cognitive load, Emotional load, and Affective load were significantly higher in away vs. home matches (*p* < 0.05). Considering season phases, in P3, Mental Fatigue was significantly higher in drawing than in losing matches (*p* < 0.05). Additionally, considering the match outcome, when the team lost, Total Distance (TD)/min and TD > 19 km·h^−^^1^/min were significantly lower in P1 than P2 (*p* < 0.001), P3 (*p* < 0.001), and P4 (*p* < 0.001). These results suggest to strength and conditioning coaches the need to consider the outcome and location of the previous game when planning the week, as well as the phase of the season they are in to reduce fatigue and injury risk.

## 1. Introduction

Soccer is an intermittent team sport characterized by the random combination of low-intensity periods (e.g., walking or jogging) with high-intensity periods (e.g., running at high speeds or sprinting) [1,2]. Specifically, professional soccer players in the Spanish First Division cover a total of 112.96 m/min, of which 6.78 m/min are completed at very high-intensity running (i.e., >21 km·h^−1^) [3]. Consequently, soccer players are exposed to high physical demands, so they must be prepared to withstand them and reduce the injury risk [4], as well as to perform as expected and increase the possibilities of success [5]. Therefore, strength and conditioning coaches, in coordination with the technical staff, should find and apply strategies to facilitate the optimal readiness of the players.

For this purpose, one of the most relevant strategies is the quantification of the training and match loads [6,7]. Workload is divided into external load, or the physical effort performed (i.e., meters or sets), and internal load [8], or the psychophysiological and psychobiological response produced by each player (i.e., mental variables or heart rate; [9]). In recent years, load quantification strategies have been difficult to implement in soccer teams, and only internal load values such as heart rate or rate of perceived exertion (RPE) were available [10]. Also, the presence of video cameras in the stadiums allowed the monitoring of the distances covered by players at different intensity levels [11]. However, the inclusion of Global Position System (GPS) devices in professional club routines has greatly facilitated this work [12], whether to improve performance [13,14], prevent injuries [15], or optimize rehabilitation programs [16]. Nevertheless, soccer is influenced by more variables than the conditional level; therefore, it seems necessary to monitor different mental variables such as mental fatigue [17] or cognitive load [18]. For example, Kunrath et al. [19] reported that mental fatigue negatively influenced players’ technical, tactical, and cognitive performance. This resulted in higher passing errors, lower shooting accuracy, and worse synchronization between players, as well as negative effects on players’ time and accuracy in decision-making. In addition, this can have an impact on players by increasing the risk of muscle injuries [20]. Despite the increase in load competition research, new studies are required to analyze both conditional and mental variables in order to achieve a more comprehensive understanding of current soccer competition demands.

Additionally, it is necessary to consider the contextual variables, such as match outcome or match location, that could affect the workload during soccer practice. For example, Andrzejewski et al. [21] analyzed the impact of match outcome on external load and observed that forwards and wide midfielders covered longer sprint distances in won matches than in lost matches. Similarly, full-backs and central defenders in the German Bundesliga covered greater distances at 21–23.99 km·h^−1^ in lost matches than win or draw matches [22]. Regarding match location (i.e., playing at home or away), playing away is associated with more total distance covered and less distance traveled at maximum intensity (>23 km·h^−1^) [23]. In this regard, the distance covered in home matches by Portuguese professional players (10,206 ± 1926 m) far exceeded that of away matches (9471 ± 1932 m). Conversely, the number of accelerations at more than 3 m·s^−2^ was higher in away matches (7 ± 5) than in home matches (6 ± 4) [24]. In the same way, Portuguese professional players covered a greater relative high-speed running distance and performed a greater number of sprints in away matches (68.62 m and 88.74 sprints) than in home matches (64.17 m and 81.32 sprints) [25]. Knowing the variables that influence match demands seems essential to help in the preparation for training and recovery strategies after each competition [26]. In addition, the influence of these variables could be different depending on the period of the season [27], so it seems necessary to analyze them.

Due to the importance of jointly quantifying mental and external load variables based on contextual variables, this study aimed to analyze the effects of contextual variables (i.e., match location and match outcome) on match load (i.e., internal and external load) in professional Brazilian soccer players. In addition, we aimed to examine these differences between season phases. We hypothesized that mental variables would be increased during away matches when the team is losing, while these demands would be greater at the end of the season, conversely to external load variables.

## 2. Materials and Methods

### 2.1. Sample

A total of thirty-six professional soccer players (M_age_ = 27.7; SD_age_ = 5.58) participated in this study. All athletes were recruited from one professional Brazilian first-level team competing in national (i.e., Mineiro Championship, Brasileirao Championship, and Brazilian Cup) and international (i.e., Libertadores Cup) tournaments during the study period. Players completed five training sessions per week (79 min on average per session) and participated in one competitive match every week (at least). Participants had 9.3 ± 5.28 years of experience playing soccer and had not reported previous injuries that limited their normal participation in the research. All field players who participated in each match (i.e., starters and non-starters) were included in the analysis. Goalkeepers were not included in the analysis due to their specific roles during the game. Before starting this research, participants signed an informed consent that explained the purposes and potential risks associated with participating in this research. The study was conducted in accordance with the Declaration of Helsinki (2013), and the protocol was approved by the ethics committee of the University of Extremadura (protocol number: 239/2019).

### 2.2. Study Design

A retrospective, descriptive, longitudinal design was applied to examine the influence of match outcome, match location, and season periods on mental, internal, and external load during official matches. All matches played during the 2022 season were included in the study (from 25 January 2022 to 12 November 2022). The team competed in four competitions during the 2022 season (Mineiro Championship, *n* = 120 players observations; Brasileiro Championship, *n* = 396 players observations; Libertadores Cup, *n* = 165 players observations; Brazilian Cup, *n* = 223 players observations). The matches were performed in official stadiums (FIFA recommendations: natural grass, ~105 × 68 m). The external load was monitored during each match, while the internal load was assessed at the end of each match.

### 2.3. Variables and Procedure

#### 2.3.1. External Load

To examine the external load of soccer players, athletes wore VectorS7s. This Global Position System (GPS) is made by Catapult Sports (Catapult Sports, Melbourne, Australia) and were positioned on each athlete in the center of their upper back. This device includes a tri-axial accelerometer, tri-axial gyroscope, and tri-axial magnetometer, each provided at sampling rates of 100 Hz, whereas GPS is sampled at 10 Hz. Similar Catapult devices and VectorS7 have previously been found to have high rates of reliability [28,29,30]. Specifically, the external load variables measured were: total distance covered by soccer players in meters per minute (TD/min), distance covered above 19 km·h^−1^ per minute (TD > 19/min); distance covered above 25 km·h^−1^ per minute (TD > 25/min); relative player load; accelerometer-derived measurements of total body load in its 3 axes (vertical, anterior-posterior, and medial-lateral; PLoad/min); the number of total high accelerations (>3 m·s^−2^; ACC) and total high decelerations (>3 m·s^−2^; DEC) performed by soccer players.

#### 2.3.2. Internal Load

Mental load perceived after matches were measured with the Questionnaire to quantify the Mental Load in Team Sports (QMLST; Questionnaire S1; [31]). The questionnaire requires players to self-report about four items: (a) Rating of Perceived Exertion (RPE; How demanding would you quantify the physical effort of this session?); (b) Cognitive load (How demanding would you quantify the cognitive effort of this session?); (c) affective load (How demanding would you quantify the effort made in this session to manage emotional relationships with the other participants?); and (d) Emotional load (How demanding would you quantify the effort made to manage your emotions during this session?). The responses ranged from 0 (no effort perceived) to 10 (maximum effort perceived). The validity and reliability of this instrument have been previously proven in soccer [32,33].

Moreover, the motivation of athletes after matches was also measured with a Visual Analogue Scale (VAS-100 mm; “How motivating does it feel to quantify the session on a line from 0 to 100?”). Players were ranked the level of motivation along a line from 0 (minimum; on the left) to 100 (maximum; on the right). Then, the original units of the VAS-100 mm for motivation were transformed to their equivalents on a scale of 0 to 10 to be consistent with the format of the other scales obtained. This scale has already been used in previous soccer studies to measure motivation [34,35,36].

Mental fatigue (MF) was assessed after official matches by a Visual Analogue Scale (VAS-100 mm). Players ranked on one single horizontal line from 0 (none at all) to 100 (maximum) to indicate how mentally fatigued they felt after the matches. This instrument has been used in previous studies exploring MF in soccer [34,35,36].

Ratio Total Distance/Mental Fatigue (RATDFM): Following the line of previous studies [37], the variable total distance/mental fatigue ratio has been generated, which simply indicates a relationship between the two variables. High values of this ratio represent a greater influence of total distance with respect to mental fatigue, while low values of this ratio indicate a greater influence of mental fatigue.

#### 2.3.3. Contextual Variables

Season phases, similar to previous research [38], every season was split into four blocks of matches to allow for easier interpretation of the data. Specifically, according to the number of matches per season, this was split into four phases: matches 1–16 (i.e., Phase 1 = P1); matches 17–32 (i.e., Phase 2 = P2); matches 33–48, (i.e., Phase 3 = P3); matches 49–65 (i.e., Phase 4 = P4).

Match location. Similar to the study by Lago-Peñas and Lago-Ballesteros [39], the match location variable identifies whether a team is playing as a Home or Away team.

Match outcome. This variable refers to the final result of the match, i.e., the difference between a win, draw, or loss [40].

### 2.4. Statistical Analysis

Data were presented as Coefficient (SE). Firstly, data normality was checked by the Kolmogorov-Smirnov test. Then, a linear mixed model (LMM) analysis using the MIXED procedure was carried out to examine the influence of match location, match outcome, and season phases on internal and external load. LMM allows us to analyze data with a hierarchical structure in nesting units and has demonstrated its ability to cope with unbalanced and repeated-measures data [41]. For example, distance covered in matches is nested for players across time (i.e., each player has a record for any match played). Consequently, a general multilevel modeling strategy was applied where fixed and random effects in different steps were included [41]. First, a two-level hierarchy was modeled for the analysis. The external load variables (i.e., distances covered at different speed thresholds) and internal load variables (i.e., mental load, mental fatigue, RPE, and motivation) were included as dependent variables in the models and match outcome (win, draw, or loss), match location (away and home), and season phases (phase 1, 2, 3, and 4) were the independent variable included as fixed effects. The player variable was considered as the random effect in the analysis. Statistical significance was set at *p* < 0.05. All statistical analyses were conducted using R-studio (version 2023.09.1+494) [42].

## 3. Results

Table 1 shows the internal load variables by match outcome and match location. Considering the match outcome, when the team drew, RPE was significantly higher in home matches vs. away matches (*p* < 0.01). In addition, when the team won, Cognitive load, Emotional load, and Affective load were significantly higher in away vs. home matches (*p* < 0.05). Considering match location, in home matches, RPE and Mental Fatigue were significantly higher in drawing matches than in losing (*p* < 0.01) and winning matches (*p* < 0.01).

Table 2 summarizes the descriptive statistics of physical variables by the match outcome and match location. Considering the match outcome, when the team lost, TD/min was significantly higher in away matches than at home (*p* < 0.05). Similarly, TD > 19 km·h^−^^1^/min was significantly higher in away matches than at home (*p* < 0.01). Considering match location, in-home matches, TD/min were significantly higher in winning than drawing matches (*p* < 0.05). On the other hand, in away matches, TD > 19 km·h^−^^1^/min was significantly higher in losing than in drawing matches (*p* < 0.05).

Table 3 shows the descriptive statistics of the mental load variables by season phases and match outcome. Considering the season phases, in P1 RPE was significantly lower in losing than drawing matches (*p* < 0.05); the cognitive and affective load was significantly higher in losing than winning matches (*p* < 0.05). In P2, there were only significant differences for mental fatigue, which was significantly higher in drawing than losing matches (*p* < 0.05). As for P3, mental fatigue was also significantly higher in drawing than losing matches (*p* < 0.05); moreover, RATDFM was significantly higher in losing than drawing and winning (*p* < 0.001) and in winning than drawing matches (*p* < 0.05). In the last phase, P4, cognitive load, emotional load, affective load, and mental fatigue were significantly lower in winning matches than in losing and drawing matches. Considering the match outcome, when the team lost, RPE was significantly lower in P1 than in P3 (*p* < 0.05); and P4 (*p* < 0.05); the cognitive load was significantly lower in P1 than in P2 (*p* < 0.001), P3 (*p* < 0.001) and P4 (*p* < 0.001); the emotional load was significantly lower in P1 than P2 (*p* < 0.001), P3 (*p* < 0.01) and P4 (*p* < 0.001); the affective load was significantly lower in P1 than P2 (*p* < 0.001), P3 (*p* < 0.05) and P4 (*p* < 0.001); motivation was significantly higher in P4 than in P2 (*p* < 0.05) and P3 (*p* < 0.05); mental fatigue was significantly higher in P4 than in P2 (*p* < 0.001) and P3 (*p* < 0.001); RATDFM was significantly lower in P1 than in P3 (*p* < 0.001) and significantly higher in P3 than in P4 (*p* < 0.001). On the other hand, when the team drew cognitive load was significantly lower in P1 than in P2 (*p* < 0.01) and P4 (*p* < 0.001). Emotional load was significantly lower in P1 than in P4 (*p* < 0.001) and in P2 than P4 (*p* < 0.05); affective load was significantly lower in P1 than in P4 (*p* < 0.001) and in P2 than P4 (*p* < 0.01); motivation was significantly lower in P1 than P4 (*p* < 0.05); mental fatigue was significantly lower in P1 than P2 (*p* < 0.05) and P4 (*p* < 0.001). Finally, when the team won, motivation was significantly lower in P1 than in P3 (*p* < 0.05); mental fatigue was significantly lower in P1 than in P4 (*p* < 0.05).

Table 4 summarizes the descriptive statistics of physical load variables by season phases and match outcome. Considering the season phases, in P1 TD/min and Pload were significantly higher in winning than drawing (*p* < 0.01). In P2 Pload was significantly higher in winning than losing (*p* < 0.01) and DEC was significantly higher in drawing than winning (*p* < 0.05). As for P3, TD/min was significantly higher in losing than drawing (*p* < 0.001) and winning (*p* < 0.05); TD > 19 km·h^−^^1^/min were significantly lower in drawing than losing (*p* < 0.01) and winning (*p* < 0.01); TD > 25 km·h^−^^1^/min was significantly lower in drawing than winning (*p* < 0.05); ACC was significantly lower in drawing than losing (*p* < 0.01) and winning (*p* < 0.05); DEC was significantly lower in drawing than losing (*p* < 0.01) and winning (*p* < 0.01). In P4 no significant differences were found in any of the variables. Considering the match outcome, when the team lost, TD/min and TD > 19 km·h^−^^1^/min were significantly lower in P1 than P2 (*p* < 0.001), P3 (*p* < 0.001) and P4 (*p* < 0.001); TD > 25 km·h^−^^1^/min were significantly lower in P1 than P2 (*p* < 0.001), P3 (*p* < 0.01) and P4 (*p* < 0.001); Pload was significantly lower in P1 than P2 (*p* < 0.01) and P3 (*p* < 0.001); DEC was significantly lower in P1 than P2 (*p* < 0.01) and P3 (*p* < 0.05). On the other hand, when the team draws, TD/min was significantly lower in P1 than P2 (*p* < 0.001) and P3 (*p* < 0.001); TD > 19 km·h^−^^1^/min was significantly lower in P1 than P2 (*p* < 0.001) and P4 (*p* < 0.001); TD > 25 km·h^−^^1^/min was significantly lower in P1 than P2 (*p* < 0.001) and P4 (*p* < 0.001); Pload was significantly higher in P1 than P2 (*p* < 0.001) and P4 (*p* < 0.001); ACC was significantly higher in P2 than P3 (*p* < 0.001); DEC was significantly higher in P2 than P3 (*p* < 0.001) and P4 (*p* < 0.01); Finally, when the team won, TD/min was significantly lower in P1 than P2 (*p* < 0.01) and P3 (*p* < 0.05); TD > 19 km·h^−^^1^/min was significantly lower un P1 (*p* < 0.001) than P2, P3 (*p* < 0.001) and P4 (*p* < 0.001); TD > 25 km·h^−^^1^/min was significantly lower in P1 than P2 (*p* < 0.01), P3 (*p* < 0.001) and P4 (*p* < 0.001); Pload was significantly lower in P1 than P2 (*p* < 0.05), and significantly higher in P2 than P3 (*p* < 0.01) and P4 (*p* < 0.01); ACC was significantly higher in P2 than P3 (*p* < 0.05) and P4 (*p* < 0.05).

## 4. Discussion

The main purpose of this study was to analyze the effects of contextual variables (i.e., match location and match outcome) on match load (i.e., mental, internal, and external load) in a professional soccer team, during a full season, considering the different periods of this season. Importantly, to our knowledge, this is the first study to explore in an integrated and ecological fashion, the changes in internal and external load indicators, promoted by contextual variables, throughout a full season. The findings of this study revealed that: (a) in won matches, the mental load is higher in away matches, (b) RPE and mental fatigue are higher in drawn than in lost or won matches, independently of match location, (c) when the beginning of the season (i.e., P1) is compared to its end (i.e., P4), in lost matches there is a significant increase in RPE, all mental load variables, motivation and mental fatigue, and (d) the same increasing pattern was observed for TD/min, TD > 19 km/h and TD > 25 km/h.

### 4.1. Internal Load Variables

In won matches, mental load is significantly affected by match location, in all its variables (i.e., cognitive, affective, and emotional load). Specifically, all the variables presented higher values in away matches when compared to home ones (Cognitive load, 7.49 vs. 7.17; Emotional load, 7.39 vs. 7.02; Affective load, 7.44 vs. 7.08). These results seem understandable, as it has been shown previously a clear relationship between increases in perceived mental load and more demanding contexts [43]; however, these higher mental load values are not consistent for all match outcomes in our study. Several authors have reported that cognitive load is related to the emotional state of the athletes and teams [44], which is sustainable with our results; in a positive context (i.e., win at home), players show lower mental load values (i.e., all variables in this study). Nevertheless, similar results should be observed for drawn and lost matches, which did not happen. Apparently, in these cases match outcome effect overlaps the match location, which indeed has been demonstrated previously [25]. Thus, it seems that the emotional burden of a non-positive match outcome (i.e., lost or draw), determines decisively the perceived mental load after the match. These results indicate that coaches should have special care in planning and managing training tasks [44]; moreover, monitoring the psychological recovery-fatigue process can be crucial to enhancing performance [45].

Players’ mental fatigue and RPE were higher in drawn than in lost or won matches, independently of match location. For example, in home matches, when the team draws, players have an RPE of 0.65 points higher than when they lose and 0.78 points higher than when they win. These are highly relevant variables to be known and controlled since mental fatigue can have physiological and behavioral consequences that affect negatively physical, technical, and decision-making performance [19,36,37,46,47]. Thus, our results sustain that a higher match outcome uncertainty (i.e., resultant from a draw), with the necessary need for a permanent alert state throughout all games [47], determines a higher perception of effort and metal fatigue. Soccer players are exposed to cognitive demands for long periods [48], independently of match results; however, in drawn matches, teams try to make a difference, particularly on the physical side [49,50], which can determine this player’s perception. Therefore, these results indicate that players and teams should be exposed to uncertain contexts in the training field, (e.g., score-wise), which will determine the development of strategies to increase cognitive, emotional, and affective abilities [33].

### 4.2. External Load Variables

Physical demands are affected by match location in lost matches, specifically with increases of 5.2% in TD/min and 16.0% in TD > 19 km/h, for teams playing away. It has been previously reported that match location has an impact on away teams’ physical performance, with higher running distances being covered in matches second half [50]. This contextual framework associated with a lost outcome can help us understand our results; as Konefal et al. [51] recently showed how to match status (i.e., losing situation) increases a team’s running distances. In soccer, home advantage is well-known and documented [52], and several factors can help determine a higher scoring ability for home teams. The psychological approach to a home match can be influenced by familiarity, territoriality, and crowd support, which can boost confidence, and influence the unique tactics and strategies developed throughout the match [53]. On the other hand, away teams often opt for or are forced to a more cautious and defensive posture [39], which may lead to longer running periods aiming to recover the ball or protect their goal. Combined with this effect, losing teams tend to increase ball possession and a sustained treat [54], aiming to create scoring goal opportunities, which underlies the need for higher running distances [55]. Importantly, for draw and win matches, our data also reports higher running distances per minute in away matches, despite no significant differences being found. These data support past research, where it has been shown that match location induces trivial changes in match-running performance [56]. However, and aiming at further research, introducing the score line state and opposition level in this analysis can add precious interpretation value; as home and away teams have different behaviors under the same positive and/or negative score [57], and the greater is the goal difference between the teams, the lower is running performance by both [49].

As for match outcome external load influence, won matches determine a higher total running distances per minute, when compared to drawn matches (TD/min home: +4.5%, away: +4.7%). Accordingly, some recent research has shown that top-ranked teams covered a significantly greater distance than lower-ranked opposition [58], which can help us understand our results, as wins are positively correlated with higher rankings. However, this has been a controversial topic since other researchers pointed in the opposite direction [58,59]. It has been argued that more organized teams do not need to run as much [59] and that losing teams tend to exhibit lower movement synchronization [60], which may lead to higher running needs. Interestingly, this assumption was partially observed in our study for high-speed running, when the loss was compared to draw (TD > 19 km/h home: +4.4%, away: +16.8%). Being aware of all the innumerable factors that could influence the running distances and intensities performed during a soccer match [61,62], these results seem to support the thesis that running performances are poor predictors of success in soccer [50,63].

### 4.3. Internal Load Variables by Season Phases and Match Outcome

#### 4.3.1. RPE and Mental Load

In the early season (i.e., P1), match outcome alters RPE, cognitive load, and affective load. Specifically, negative scores induced a lower RPE (−5.7%), when compared with drawn matches. These results confirm Fessi and Moalla’s [63] observations, with RPE being negatively affected by lost matches. Furthermore, RPE increases significantly in lost matches for the later stages of the competition (i.e., P3 and P4) when compared with the earliest ones. These increases may be related to players covering higher distances in mid-season periods [27], and/or accumulated seasonal fatigue. Irrespective of this, RPE results interpretation should be taken carefully; research has demonstrated that session RPE is not linked to the in-session RPE [64], meaning that the specific effort conducted throughout the match can be biased by the final match outcome.

Regarding mental load variables, during P1 lower values were found in lost matches, for cognitive load (−7.5%), and affective load (−5.0%), when compared with win matches. Interestingly, these differences were not observed when the full season was analyzed, being specific from this early-season period. RPE, cognitive, and affective load variables indicate that at the start of the season, perceived physical and mental effort is closely related to match outcome. This can be resulting from two main reasons: (a) evidence shows a strong influence of the first matches in a competition final ranking [65], which can introduce an extra contextual variable, determined by the need for a premature high sport form; and (b) being the cognitive load closely related to the emotional state of the players [44], and representing the amount of mental resources invested to solve problems and tasks [66], the start of a competition determines higher levels of individual and collective uncertainty, which may arouse several emotional states and lead to higher values of perceived mental and physical effort, particularly when more demanding tasks are accomplished (i.e., winning). Comparing season phases, it is interesting to observe that in lost and drawn matches, the cognitive, emotional, and affective load is always lower in the early season (P1), when compared with the end of the season (P4). This growing mental demand is coherent with Teixeira et al. [25] conclusions, reinforcing the relevance of match outcome over other contextual variables, which can be particularly important with the approach of the end of the season. Additionally, these results reinforce the relevance of the individual emotional interpretation and response to specific events (i.e., match outcome) [67]; also, these emotions can be resultant from the exposure to events with special relevance to the group [68]. Irrespective of this, no mental load changes were observed in any of the variables, throughout the season, in won matches. This means that in positive contexts, the emotional and cognitive effort, defined as the selection and use of resources to respond to the demands of a specific task [69], remains stable throughout the season, which may favor the coach’s planning and management.

#### 4.3.2. Motivation, Mental Fatigue, and Ratio Total Distance and Mental Fatigue

Inside each season phase, motivation values remain unchanged between different contextual scenarios (i.e., lost, draw, and win). However, the same is not true when phases are compared, and it is possible to observe an increasing trend in all scenarios, particularly between P1 and P4, for losing and drawing outcomes. Motivation levels in soccer, as in any other sport, depend on individual and contextual factors [70]; additionally, the ability of players to remain motivated, even in situations of uncertainty and pressure, can help them perform [71]. By these means, the fact that players reveal unchanged levels of motivation inside season periods, independently of match outcomes, indicates these players’ motivation continuum seems to result fundamentally from intrinsic reasons [72], which can be positively observed in seasonal competition management.

Regarding mental fatigue, it has been shown that this variable can alter significantly decision-making abilities, perception, attention [73], or even technical performance in soccer [74]. Conversely, other studies did not find a relationship between mental fatigue and technical performance in players [36]. Being aware of these inconsistent results, and also knowing mental fatigue is a complex psychophysiological phenomenon, it is important to understand its dynamics in a full-season period, and how it can affect an also complex and multifactorial sport, like soccer. In our study, during mid-season (i.e., P2 and P3), mental fatigue grows in drawn, compared with lost matches. Moreover, at the end of the season period (i.e., P4), drawn matches induced not only higher levels of mental fatigue, compared to lost, but also to won matches. This growing value in undefined results (i.e., draw) is quite interesting, meaning that, drawing specific conditions by inducing a demanding increase for cognitive tasks, may lead to mental fatigue [75]. The same behavior is observed between the early season (i.e., P1) and the end of the season (i.e., P4). A long and demanding competitive season, is a special and almost unique characteristic of soccer, meaning players in this sport are under a longer fatiguing stimulus, which seems to lead players to a status where physical, technical, and tactical issues may be impaired, inside competition contexts. Intriguingly, it has been proposed that mental fatigue can be promoted by the increase of adenosine, which may lead to decreases in the perception of effort and motivation [47], which contradicts our results. Throughout the season there is a growing effect on both motivation and mental fatigue, something that should be explored in further research, particularly with the use of different instruments and/or scales.

The ratio of total distance and metal fatigue presents differences between season phases, specifically in lost matches, where phase 3 presents higher values than in any other seasonal period. Since mental fatigue only significantly increases in phase 4, these values indicate that at the end of the mid-season period, there is an unbalanced effect between a growing physical demand and an unchanged mental impact; this trend is corrected in the final period of the season (i.e., P4), with growing mental fatigue and a decreasing running distance behavior (i.e., between P3 and P4). These results seem to reinforce the combined consequence between a lost matches effect [51], and a better physical availability in the mid-season [27]; which interestingly is also observed in a shorter window period (i.e., P3). The fact that lost matches increase this ratio, and present significantly higher values than drawn (i.e., +84.8%) or won (i.e., +26.7%) at P3, adds strength to these conclusions.

### 4.4. External Load Variables by Season Phases and Match Outcome

In the early season, there are punctual differences between drawn and won matches, particularly for total TD/min and player load. Despite our results demonstrating that running more distance and having more accelerations and decelerations differentiates won from drawn matches in this specific season period, their exceptional character reinforces the need to not overestimate the importance of running as a success predictor [49]. Nevertheless, by analyzing mid-season data, deeper insight and discussion are needed, as drawn matches determine lower TD/min, TD > 19 km/h, number of accelerations and decelerations, than lost or won matches; and lower TD > 25 km/h than lost matches. This more consistent pattern indicates that players cover lower running distances and have less accelerations-decelerations, when the match outcome is more uncertain (i.e., draw), and teams seem more stable. Interestingly, our findings for high-intensity running distances are in line with the trend shown by Teixeira et al. [25] in the Portuguese Professional League, where drawing situations also lead to lower distances covered in high-speed running and sprinting, when compared to losing or winning. Finally, it is important to highlight that there are significant differences between early season running distances (i.e., TD/min, TD > 19 km/h, and TD > 25 km/h), and the following ones (i.e., P2, P3 and P4), independently of match outcome, which demonstrates increases in RPE, mental load and mental fatigue does not determine a decrement in the physical ability of the players; indeed, it can reinforce Kunrath et al. [19] observations, indicating that mental fatigue can damage the efficacy of the tactical behavior of the players, and that can be somehow compensated by an increase in the physical aspects of the game. On the other hand, knowing that running performance is altered by the quality of the opposition [25], and is position-dependent [22,24], suggests these factors must be deeply explored for a better comprehension of the results in this specific topic.

### 4.5. Limitations

This study presents some limitations that must be considered. Firstly, a single team was analyzed, so specific variables such as classification, team formation, or coach style could have influenced the obtained results. Secondly, other contextual variables could affect the results obtained, such as the quality of the opponent. Therefore, it is a variable to be considered for future studies. Thirdly, only male participants were involved in this study, so the obtained findings must be cautiously extrapolated to female populations. Finally, the Brazilian league presents differences from European leagues, so further studies in other countries and also involving female soccer players must be conducted to establish a more robust conclusion on this topic.

## 5. Conclusions

This study has obtained relevant findings which must be highlighted: (i) in won matches, the mental load is higher in away matches, (ii) RPE and mental fatigue are higher in draw matches than in lost or won, independently of match location, (iii) when the beginning of the season (i.e., P1) is compared to its end (i.e., P4), in lost matches there is a significant increase in RPE, all mental load variables, motivation and mental fatigue, and (iv) the same increasing pattern was observed for TD/min, TD > 19 km/h and TD > 25 km/h. In practical terms, strength and conditioning coaches should pay special attention to mental load management during away matches, as well as consider recovery strategies specifically tailored to address the unique demands of draw matches. In addition, the results of the study suggest the necessity for periodized training programs aimed to progressively increase the intensity of training sessions. Moreover, providing psychological support is essential to help players adapt to the changing demands and stressors throughout the season.

## Figures and Tables

**Table 1:** Differences in internal load variables by match location and match outcome.

	Lost					Draw					Win						
	Home		Away		*p*	Home		Away		*p*	Home		Away		*p*	*p* (Home)	*p* (Away)
	Coeff	SE	Coeff	SE		Coeff	SE	Coeff	SE		Coeff	SE	Coeff	SE			
RPE	7.48	0.18	7.56	0.17	0.636	8.13	0.21	7.50	0.20	**	7.35	0.16	7.64	0.19	0.071	a **, c **	
Cognitive load	7.30	0.21	7.46	0.20	0.334	7.41	0.23	7.55	0.23	0.526	7.17	0.19	7.49	0.22	*		
Emotional load	7.32	0.21	7.42	0.20	0.537	7.39	0.23	7.34	0.22	0.791	7.02	0.19	7.39	0.22	*		
Affective load	7.35	0.20	7.51	0.20	0.316	7.47	0.23	7.41	0.22	0.770	7.08	0.19	7.44	0.21	*		
Motivation	7.82	0.25	7.95	0.24	0.663	8.06	0.31	7.76	0.30	0.447	7.85	0.20	7.88	0.27	0.901		
Mental Fatigue	72.2	1.65	71.0	1.61	0.478	77.1	1.98	73.7	1.87	0.120	69.3	1.44	70.9	1.77	0.323	a **, c **	
RATDMF	85.5	4.86	91.1	4.71	0.288	87.3	5.96	83.0	5.60	0.538	95.2	4.13	88.9	5.26	0.212		

Note. Coeff = Coefficient; SE = Standard Error; RPE = Rating of perceived exertion; RATDMF = Ratio between Total Distance and Mental Fatigue; a = significant differences between lost vs. draw; c = significant differences between draw vs. win; * *p* < 0.05; ** *p* < 0.01.

**Table 2:** Differences in external load variables by march location and match outcome.

	Lost					Draw					Win						
	Home		Away		*p*	Home		Away		*p*	Home		Away		*p*	*p* (Home)	*p* (Away)
	Coeff	SE	Coeff	SE		Coeff	SE	Coeff	SE		Coeff	SE	Coeff	SE			
TD (m/min)	89.3	4.21	93.9	4.19	*	88.7	4.37	90.0	4.32	0.597	92.3	4.11	94.2	4.27	0.293	c *	
TD > 19 km·h^−1^ (m/min)	6.10	0.47	7.08	0.46	**	5.83	0.51	5.89	0.50	0.892	6.41	0.44	6.55	0.49	0.673		a *
TD > 25 km·h^−1^ (m/min)	1.29	0.14	1.43	0.14	0.256	1.36	0.16	1.18	0.16	0.286	1.35	0.13	1.32	0.15	0.839		
PLoad (m/min)	10.1	0.60	10.5	0.59	0.245	10.3	0.63	10.2	0.62	0.780	10.4	0.58	10.6	0.61	0.570		
ACC (nº.)	21.6	1.24	20.9	1.22	0.545	19.4	1.43	20.9	1.37	0.311	20.3	1.13	20.1	1.31	0.837		
DEC (nº.)	25.5	1.67	25.6	1.64	0.952	24.6	1.86	24.5	1.80	0.982	25.0	1.55	23.8	1.74	0.342		

Note. Coeff = Coefficient; SE = Standard Error; ACC = Accelerations > 3 m·s^−2^; DEC = Decelerations > 3 m·s^−2^; a = significant differences between lost vs. draw; c = significant differences between draw vs. win; * *p* < 0.05; ** *p* < 0.01.

**Table 3:** Differences in internal load variables by season phases and match outcome.

		P1			P2			P3			P4			*p*
		*Coeff*	*SE*	*p*	*Coeff*	*SE*	*p*	*Coeff*	*SE*	*p*	*Coeff*	*SE*	*p*	
RPE	Lost	7.08	0.17	#*	7.35	0.18		7.60	0.24		7.64	0.21		b *, c *
	Drawn	7.51	0.19		7.65	0.21		7.01	0.43		7.89	0.30		
	Won	7.49	0.22		7.35	0.19		7.24	0.17		7.31	0.19		
Cognitive load	Lost	6.65	0.19	$*	7.36	0.19		7.48	0.25		7.95	0.22	$**, ♦**	a ***, b ***, c ***, e **
	Drawn	6.93	0.20		7.60	0.22		7.48	0.43		8.34	0.31		a **, c ***, e *
	Won	7.19	0.23		7.27	0.20		7.37	0.19		7.35	0.20		
Emotional load	Lost	6.67	0.18		7.41	0.19		7.31	0.24		7.78	0.22	$*, ♦**	a ***, b **, c ***
	Drawn	7.02	0.20		7.45	0.22		7.56	0.42		8.12	0.30		c ***, e *
	Won	6.96	0.22		7.28	0.20		7.34	0.18		7.25	0.20		
Affective load	Lost	6.88	0.18	$*	7.43	0.19		7.24	0.24		8.06	0.22	$***, ♦***	a ***, b *, c ***, e **, f **
	Drawn	7.01	0.20		7.43	0.22		7.55	0.42		8.29	0.30		c ***, e **
	Won	7.24	0.22		7.26	0.20		7.27	0.18		7.28	0.20		
Motivation	Lost	7.24	0.23		7.75	0.24		7.46	0.35		8.53	0.30		c ***, e *, f *
	Drawn	7.46	0.27		7.76	0.30		7.77	0.67		8.66	0.46		c *
	Won	7.34	0.35		7.66	0.26		8.14	0.23		7.96	0.26		b *
Mental Fatigue	Lost	64.2	1.69		67.3	1.75	#*	65.0	2.33	#*	78.8	2.07	$**, ♦***	c ***, e ***, f ***
	Drawn	67.7	1.87		72.6	2.07		75.2	4.23		83.2	2.97		a *, c ***, e **
	Won	66.3	2.15		68.5	1.87		68.6	1.67		71.4	1.86		c *
RATDFM	Lost	89.3	4.82		92.9	4.99		116.4	6.84	#***, $***, ♦*	76.5	6.02		b ***, d **, e *, f ***
	Drawn	86.3	5.41		87.7	6.03		63.0	12.67		76.0	8.81		
	Won	90.2	6.27		89.4	5.38		91.9	4.74		85.6	5.35		

Note. Coeff = Coefficient; SE = Standard Error; RPE = Rating of perceived exertion; RATDMF = Ratio between Total Distance and Mental Fatigue; P1 = Phase 1; P2 = Phase 2; P3 = Phase 3; P4 = Phase 4; # = significant differences between −1 and 0; $ = significant differences between −1 and 1; ♦ = significant differences between 0 and 1; a = significant differences between P1 and P2; b = significant differences between P1 and P3; c = significant differences between P1 and P4; d = significant differences between P2 and P3; e = significant differences between P2 and P4; f = significant differences between P3 and P4. * *p *< 0.05, ** *p* < 0.01, *** *p* < 0.001.

**Table 4:** Differences in external load variables by season phases and match outcome.

		P1			P2			P3			P4			*p*
		*Coeff*	*SE*	*p*	*Coeff*	*SE*	*p*	*Coeff*	*SE*	*p*	*Coeff*	*SE*	*p*	
TD (m/min)	Lost	74.4	4.17	♦**	87.7	4.24		96.6	4.88	#***, $*, ♦*	89.8	4.59		a ***, b ***, c ***, d *
	Drawn	69.1	4.35		92.9	4.58		72.2	7.34		93.2	5.65		a ***, c ***, d **, f **
	Won	80.0	4.65		90.1	4.36		89.2	4.17		86.8	4.36		a **, b *
TD > 19 km·h^−1^ (m/min)	Lost	4.10	0.48		6.54	0.49		6.72	0.58	#**, ♦**	7.31	0.54		a ***, b ***, c ***
	Drawn	4.08	0.50		6.35	0.54		3.79	0.90		7.11	0.68		a ***, c ***, d **, f ***
	Won	4.20	0.54		6.34	0.50		6.39	0.48		6.49	0.50		a ***, b ***, c ***
TD > 25 km·h^−1^ (m/min)	Lost	0.84	0.14		1.28	0.14		1.34	0.18	♦*	1.65	0.16		a ***, b **, c ***, e *
	Drawn	0.76	0.15		1.50	0.16		0.78	0.30		1.66	0.22		a ***, c ***, d *, f *
	Won	0.70	0.17		1.23	0.15		1.40	0.14		1.46	0.15		a **, b ***, c ***
PLoad (m/min)	Lost	8.59	0.54	♦**	9.78	0.55	$**	10.52	0.64		9.45	0.60		a **, b ***
	Drawn	7.98	0.56		10.55	0.60		9.38	0.99		10.30	0.75		a ***, c ***
	Won	9.53	0.61		10.75	0.57		9.54	0.54		9.47	0.57		a *, d **, e **
ACC (n.º)	Lost	21.0	1.18		22.4	1.22		20.9	1.57	#**, ♦*	20.4	1.45		
	Drawn	19.6	1.29		22.4	1.41		12.7	2.76		22.5	1.96		b *, d ***, f **
	Won	20.7	20.7		22.5	1.29		19.6	1.17		19.3	1.29		d *, e *
DEC (nº.)	Lost	21.6	1.57		26.0	1.60	♦*	25.6	1.99	#**, ♦**	24.5	1.85		a **, b *
	Drawn	21.5	1.68		28.8	1.81		14.6	3.32		22.3	2.42		a ***, b *, d ***, e **, f *
	Won	24.0	1.86		25.2	1.68		23.9	1.56		23.4	1.68		

Note. Coeff = Coefficient; SE = Standard Error; ACC = Accelerations > 3 m·s^−2^; DEC = Decelerations > 3 m·s^−2^; P1 = Phase 1; P2 = Phase 2; P3 = Phase 3; P4 = Phase 4; # = significant differences between −1 and 0; $ = significant differences between −1 and 1; ♦ = significant differences between 0 and 1; a = significant differences between P1 and P2; b = significant differences between P1 and P3; c = significant differences between P1 and P4; d = significant differences between P2 and P3; e = significant differences between P2 and P4; f = significant differences between P3 and P4; * *p *< 0.05, ** *p* < 0.01, *** *p* < 0.001.

## Data Availability

Data are available upon request to the corresponding author.

## Supplementary Materials

The following supporting information can be downloaded at: https://www.mdpi.com/article/10.3390/s24020679/s1, Questionnaire S1: Questionnaire to quantify the Mental Load in Team Sports (QMLST).
