# Supplementary material for: Effects of Contextual Variables on Match Load in a Professional Soccer Team Attending to the Different Season Periods
Source: Sensors (Basel). 2024 Jan 21;24(2):679. doi: 10.3390/s24020679 (PMC10820192; doi:10.3390/s24020679)
Supplement: Supplementary file 1 [file sensors-24-00679-s001.zip › sensors-2819112-supplementary.pdf]

Questionnaire S1. Questionnaire to quantify the Mental Load in Team Sports (QMLST)

	**0**	**1**	**2**	**3**	**4**	**5**	**6**	**7**	**8**	**9**	**10**
**RPE**	Rest	Very, very easy	Easy	Moderate	Somewhat hard	Hard		Very hard			Maximal
**COGNITIVE LOAD**	No effort perceived										Maximun effort perceived
**AFFECTIVE LOAD**	No effort perceived										Maximun effort perceived
**EMOTIONAL LOAD**	No effort perceived										Maximun effort perceived
